# Supplementary material for: Finite blocklength feedback approach for multi-antenna wireless channels
Source: PLoS One. 2024 Feb 15;19(2):e0297810. doi: 10.1371/journal.pone.0297810 (PMC10868777; doi:10.1371/journal.pone.0297810)
Supplement: Attachment — Submitted filename: Response-to-Reviewers.pdf [file pone.0297810.s001.pdf]

Response to Referee Report on PONE-D-23-29974: *Finite Blocklength Feedback Approach for Multi-Antenna Wireless Channels*

Qiang Huang, and Tao Song

We would like to express our sincere gratitude to the editor and all the reviewers for a thorough review with detailed and valuable comments. The raised comments are very helpful for improving the quality of the manuscript. We have addressed all the comments with utmost carefulness. By taking full account of the comments, we made some changes to the manuscript. The reply to the editor and reviewers' comments is attached below.

Reply to Editor:

Q1) After careful consideration, we feel that it has merit but does not fully meet PLOS ONE's publication criteria as it currently stands. Therefore, we invite you to submit a revised version of the manuscript that addresses the points raised during the review process.

Answer: Thank you for giving us this chance to update our manuscript. Based on the reviewers' suggestions, the revision of this manuscript mainly includes:

- According to Reviewer 1's suggestion, we revised Introduction of this paper by adding the literature review of finite blockleng coding.
- As suggested by the reviewers, in the revised manuscript, we revise the motivation of this paper.
- In the revised manuscript, the results given in Theorems 1-4 are re-expressed in terms of Shannon capacity, as suggested by Reviewer 2.
- Based on Reviewer 3's suggestion, we added the assumptions in the manuscript, i.e., the channel state information (CSI) stays constant during the entire transmission and it's shared by the transceiver.
- As the reviewers suggested, we checked through the manuscript and corrected several typos.

Reply to Reviewer 1:

Q1) When introducing the FBL analyses, the authors ignored the work of Strassen [1] and downgraded the work of Hayashi [2, 3]. In fact, second-order asymptotics was first studied by Strassen and first revived by Hayashi using information spectrum methods [4]. The notion of "second-order coding rate" was also coined by Hayashi. Furthermore, the equation in the FBL analysis is not numbered and the third-order term has been characterized for certain channels. The authors could refer to [5, 6] for details on FBL analyses and history.

Answer: Thank you very much for the comment! As suggested by the reviewer, we add literature review of the finite blocklength coding in Introduction, and for convenience, we attach it below.

The analysis of FBL coding receives a great deal of attention. To be specific, the second-order asymptotics for discrete memoryless case was first studied by Strassen [1]. Then Hayashi [2]-[3] extended the result in [1] to more general cases by using the information spectrum method [4], and obtained the optimum second-order coding rate [2]. In addition, [5] presented a unified treatment for asymptotic estimates in information theory with non-vanishing error probabilities, and [6] provided a comprehensive review of recent advances in second-order asymptotics for lossy source coding.

Q2) The definition of the fundamental limit in (4) is incomplete. It only specifies the achievability part. However, a formal definition should specify the optimal rate, as in [7, 8].

Answer: Thank you very much for the comment! In the revised manuscript, we added the maximal achievable rate and the fundamental limit, which is attached below.

The (n, ε)-transmission rate $R(n, \varepsilon)$ is achievable if for given blocklength n and error probability ε, there is a $(n, |\mathcal{W}|, P)$-code introduced in Definition 1 such that

$$\frac{\log|\mathcal{W}|}{n} = R(n, \varepsilon), \quad P_e \leq \varepsilon, \tag{1}$$

and the maximal rate $R^*(n, \varepsilon)$ is the maximum rate defined in (1). In addition, the capacity is defined as

$$C = \lim_{N\to\infty} \lim_{\varepsilon\to 0} R^\star(n, \varepsilon). \tag{2}$$

Q3) What is the significance of Theorem 1 and why is it important? Compared with [8], the results appear in very different forms. What is the intuition here and why is it reasonable. Furthermore, since Theorem 1 is only an achievability result, what is the converse bound? How large is the gap? All these questions are critical for finite blocklength analyses. Finally, the proof of Theorem 1 appears rather strange in the perspective of finite blocklength analyses. The above comments for Theorem 1 apply to all other theorems in the paper.

Answer: Thank you very much for the comment!

- (1) Significance and importance: Theorem 1 shows an achievable bound for the SISO channel with noiseless feedback in the FBL regime, which is based on a special class of linear feedback scheme known as the classical Schalkwijk-Kailath (SK) scheme. Note that the achievable bound in Theorem 1 is the upper bound on the results of any linear feedback coding schemes for the SISO case. The study in this paper provides a new method for FBL coding in wireless systems.
- (2) The reason for different terms compared with [8]: We re-express all theorems by using the form of Shannon limit in the revised manuscript, which is attached below. Since the proposed SK-type coding scheme is a deterministic coding scheme, the subtracted part in our result is the concrete loss caused by the given codeword length and decoding error probability, instead of the term of second-order rate in [8].
- (3) About the converse bound: Honestly, the converse bounds in FBL regime for multi-antenna systems with noiseless feedback are challenging for us, which will be our future work. Instead of giving such FBL converse bounds, in this paper, the classical Shannon capacities of SISO/SIMO/MISO/MIMO channels are given as the upper bounds on the FBL capacities. The above statements (1)-(3) also applies to Theorems 2-4.

Theorem 1. For given ε and n, an achievable rate $R_{siso}(n,\varepsilon)$ for the SISO channel with noiseless feedback link is given by

$$R_{siso}(n,\varepsilon) = C_{siso} - \underbrace{\frac{1}{n}\log\left[\frac{1}{3}(Q^{-1}(\frac{\varepsilon}{4}))^2\left(\frac{\sigma^2}{P|h_{siso}|^2}+1\right)\right]}_{\textbf{Loss caused by given } (n,\varepsilon)}, \tag{3}$$

where $C_{siso} = \log\left(1+\frac{|h_{siso}|^2 P}{\sigma^2}\right)$ is the capacity of the SISO channel [9]. Since feedback does not increase the capacity of the memoryless channels, C_{siso} is also the capacity of the SISO channel with noiseless feedback, which serves as an upper bound on $R_{siso}(n,\varepsilon)$.

Theorem 2. For given ε and n, an achievable rate $R_{simo}(n,\varepsilon)$ for the SIMO channel with noiseless feedback link is given by

$$R_{simo}(n,\varepsilon) = C_{simo} - \underbrace{\frac{1}{n}\log\left[(Q^{-1}(\frac{\varepsilon}{4}))^2\left(\frac{\sigma^2}{3P||\mathbf{h}_{simo}||^2}+\frac{1}{3}\right)\right]}_{\textbf{Loss caused by given } (n,\varepsilon)}, \tag{4}$$

where $C_{simo} = \log\left(1+\frac{||\mathbf{h}_{simo}||^2 P}{\sigma^2}\right)$ is the capacity of the SIMO channel [9]. Since feedback does not increase the capacity of the memoryless channels, C_{simo} is also the capacity of the SIMO channel with noiseless feedback, which serves as an upper bound on $R_{simo}(n,\varepsilon)$.

Theorem 3. For given ε and n, an achievable rate $R_{miso}(n,\varepsilon)$ for the MISO channel with noiseless

feedback is given by

$$R_{miso}(n,\varepsilon)=C_{miso}-\underbrace{\frac{1}{n}\log\left[(Q^{-1}(\frac{\varepsilon}{4}))^2\left(\frac{\sigma^2}{3P||\mathbf{h}_{miso}||^2}+\frac{1}{3}\right)\right]}_{\textbf{Loss caused by given } (n,\varepsilon)}, \tag{5}$$

where $C_{miso}=\log\left(1+\frac{||\mathbf{h}_{miso}||^2P}{\sigma^2}\right)$ is the capacity of the MISO channel [9]. Since feedback does not increase the capacity of the memoryless channels, C_{miso} is also the capacity of the MISO channel with noiseless feedback, which serves as an upper bound on $R_{miso}(n,\varepsilon)$.

Theorem 4. For given ε and n, an achievable rate $R_{mimo}(n,\varepsilon)$ for the MIMO channel with noiseless feedback is given by

$$R_{mimo}(n,\varepsilon)=C_{mimo}-\underbrace{\min_{\sum_{k=1}^{K}P_k=P}\frac{1}{n}\sum_{k=1}^{K}\log\left[\left(\frac{\sigma^2}{3P_kd_k^2}+\frac{1}{3}\right)\left(Q^{-1}\left(\frac{\varepsilon}{4}\right)\right)^2\right]}_{\textbf{Loss caused by given } (n,\varepsilon)}, \tag{6}$$

where $C_{mimo}=\max_{\sum_{k=1}^{K}P_k=P}\sum_{k=1}^{K}\left[\log\left(1+\frac{d_k^2P_k}{\sigma^2}\right)\right]$ is the capacity of the MIMO channel [9]. Since feedback does not increase the capacity of the memoryless channels, C_{mimo} is also the capacity of the MIMO channel with noiseless feedback, which serves as an upper bound on $R_{mimo}(n,\varepsilon)$.

Q4) The novelty of the paper is unclear to me because the analysis is not proved tight or connected with any prior art in the same direction. The analyses appear strange to me.

Answer: Thank you very much for the comment! We restated the innovations in the revised manuscript, which is attached below.

The classical SK coding scheme has been proved to be an excellent FBL scheme in [10]. The basic intuition of the classical SK is: at each time instant, the receiver does minimum mean square estimation about the transmitted message, and sends his estimation back to the transmitter via a noiseless feedback channel. Since the transmitter knows the real message, the receiver's estimation error in the last time is known by the transmitter. Then in the next time, the transmitter sends the receiver's estimation error in the last time to the receiver via the AWGN channel. It has been shown that the decoding error probability of the classical SK scheme double-exponentially decays as the codeword length increases, which indicates that to achieve a desired decoding error probability, the codeword length of the SK scheme is significantly short. However, the classical SK coding scheme is mainly investigated in point-to-point AWGN channel, and this paper aims to extend to the classical SK scheme to SISO/SIMO/MISO/MIMO channels. The technical innovations are given below:

⋄ For the SISO channel: Since the channel parameters are complex-valued number, by using channel splitting, we divide the SISO channel into two equivalent sub-channels with real-valued channel parameter. Then, applying the classical SK scheme to each of the sub-channels, the SK-type FBL scheme for the SISO channel is obtained.

◇ For the SIMO channel: For the SIMO channel, since the receiver is equipped with multiple antennas, the SIMO channel can be transformed into SISO channel by using receiving beamforming technique. Then applying the SK-type FBL scheme for SISO channel to the obtained SISO channel, the SK-type FBL scheme for the SIMO channel is obtained.

◇ For the MISO channel: For the MISO channel, since the transmitter is equipped with multiple antennas, the MISO channel can be transformed into SISO channel by using precoding technique. Then applying the SK-type FBL scheme for SISO channel to the obtained SISO channel, the SK-type FBL scheme for the MISO channel is obtained.

◇ For the MIMO channel: For the MIMO channel, since the channel gain is a matrix, the MIMO channel can be transformed into multiple parallel sub-channels by singular value decomposition technique. Then applying the SK-type FBL scheme for SISO channel to the each SISO sub-channel, the SK-type FBL scheme for the MIMO channel is obtained.

Q5) Minor comments: 1) The abbreviations SISO/SIMO/MISO/MIMO appear all undefined. 2) The additional space after the dash between two references are unnecessary. 3) In code definition, the parameter comes first and the phrase “code” appears after (cf. [7]). 4) The non-math contents in math equations including “Pr” and “SISO” should be in the mathrm command so that they are italic in math environment. 5) The bracket in Eq. (6) (and other equations) is not large enough.

Answer: Thank you very much for the comment! We have tried our best to check through the manuscript and carefully corrected the issues mentioned above.

References:

[1] V. Strassen, “Asymptotische abschätzungen in shannon’s informationstheorie,” in Trans. Third Prague Conf. Information Theory, 1962, pp. 689-723.

[2] M. Hayashi, “Information spectrum approach to second-order coding rate in channel coding,” IEEE Trans. Inf. Theory, vol. 55, no. 11, pp. 4947-4966, 2009.

[3] M. Hayashi, “Second-order asymptotics in fixed-length source coding and intrinsic randomness,” IEEE Trans. Inf. Theory, vol. 54, no. 10, pp. 4619-4637, 2008.

[4] T. S. Han, Information-Spectrum Methods in Information Theory. Springer Berlin Heidelberg, 2003.

[5] V. Y. F. Tan, “Asymptotic estimates in information theory with non-vanishing error probabilities,” Foundations and Trends in Communications and Information Theory, vol. 11, no. 1-2, pp. 1-184, 2014.

[6] L. Zhou and M. Motani, “Finite blocklength lossy source coding for discrete memoryless sources,” Foundations and Trends in Communications and Information Theory, vol. 20, no. 3, pp.

157-389, 2023.

[7] Y. Polyanskiy, H. V. Poor, and S. Verdú, "Channel coding rate in the finite blocklength regime," IEEE Trans. Inf. Theory, vol. 56, no. 5, pp. 2307-2359, 2010.

[8] Y. Polyanskiy, H. V. Poor, and S. Verdú, "Feedback in the non-asymptotic regime," IEEE Trans. Inf. Theory, vol. 57, no. 8, pp. 4903-4925, 2011.

[9] D. Tse, and P. Viswanath, Fundamentals of wireless communication, Cambridge university press, 2005.

[10] L. V. Truong, S. L. Fong and V. Y. F. Tan, "On Gaussian channels with feedback under expected power constraints and with non-vanishing error probabilities," IEEE Trans. Inf. Theory, vol. 63, no. 3, pp. 1746-1765, 2017.

Reply to Reviewer 2:

The authors have introduced constructive SK-type FBL feedback coding schemes for wireless fading channels. Simulation results show that the proposed feedback-based schemes almost approach the SISO/MISO/SIMO/MIMO channel capacities with short blocklength. I consider that the manuscript shows relevant and well described findings in terms of the FBL coding scheme for the wireless multi-antenna fading channels. Overall, it is well-written and comprehensible. I have a few minor suggestions based on my review:

Q1) Can the results of this paper (Theorem 1-Theorem 4) be expressed in terms of Shannon capacity, as in Polyanskiy et al.?

Answer: Thank you very much for the comment! In the revised manuscript, we re-wrote Theorem 1-Theorem 4 as the reviewer suggested.

Theorem 1. For given ε and n, an achievable rate $R_{siso}(n,\varepsilon)$ for the SISO channel with noiseless feedback link is given by

$$R_{siso}(n,\varepsilon) = C_{siso} - \underbrace{\frac{1}{n}\log\left[\frac{1}{3}(Q^{-1}(\frac{\varepsilon}{4}))^2\left(\frac{\sigma^2}{P|h_{siso}|^2}+1\right)\right]}_{\textbf{Loss caused by given } (n,\varepsilon)}, \tag{7}$$

where $C_{siso} = \log\left(1+\frac{|h_{siso}|^2 P}{\sigma^2}\right)$ is the capacity of the SISO channel [1]. Since feedback does not increase the capacity of the memoryless channels, C_{siso} is also the capacity of the SISO channel with noiseless feedback, which serves as an upper bound on $R_{siso}(n,\varepsilon)$.

Theorem 2. For given ε and n, an achievable rate $R_{simo}(n,\varepsilon)$ for the SIMO channel with noiseless

feedback link is given by

$$R_{simo}(n,\varepsilon) = C_{simo} - \underbrace{\frac{1}{n}\log\left[(Q^{-1}(\frac{\varepsilon}{4}))^2\left(\frac{\sigma^2}{3P||\mathbf{h}_{simo}||^2}+\frac{1}{3}\right)\right]}_{\textbf{Loss caused by given } (n,\varepsilon)}, \tag{8}$$

where $C_{simo} = \log\left(1+\frac{||\mathbf{h}_{simo}||^2 P}{\sigma^2}\right)$ is the capacity of the SIMO channel [1]. Since feedback does not increase the capacity of the memoryless channels, C_{simo} is also the capacity of the SIMO channel with noiseless feedback, which serves as an upper bound on $R_{simo}(n,\varepsilon)$.

Theorem 3. For given ε and n, an achievable rate $R_{miso}(n,\varepsilon)$ for the MISO channel with noiseless feedback is given by

$$R_{miso}(n,\varepsilon) = C_{miso} - \underbrace{\frac{1}{n}\log\left[(Q^{-1}(\frac{\varepsilon}{4}))^2\left(\frac{\sigma^2}{3P||\mathbf{h}_{miso}||^2}+\frac{1}{3}\right)\right]}_{\textbf{Loss caused by given } (n,\varepsilon)}, \tag{9}$$

where $C_{miso} = \log\left(1+\frac{||\mathbf{h}_{miso}||^2 P}{\sigma^2}\right)$ is the capacity of the MISO channel [1]. Since feedback does not increase the capacity of the memoryless channels, C_{miso} is also the capacity of the MISO channel with noiseless feedback, which serves as an upper bound on $R_{miso}(n,\varepsilon)$.

Theorem 4. For given ε and n, an achievable rate $R_{mimo}(n,\varepsilon)$ for the MIMO channel with noiseless feedback is given by

$$R_{mimo}(n,\varepsilon) = C_{mimo} - \underbrace{\min_{\sum_{k=1}^{K}P_k=P}\frac{1}{n}\sum_{k=1}^{K}\log\left[\left(\frac{\sigma^2}{3P_k d_k^2}+\frac{1}{3}\right)\left(Q^{-1}\left(\frac{\varepsilon}{4}\right)\right)^2\right]}_{\textbf{Loss caused by given } (n,\varepsilon)}, \tag{10}$$

where $C_{mimo} = \max_{\sum_{k=1}^{K}P_k=P}\sum_{k=1}^{K}\left[\log\left(1+\frac{d_k^2 P_k}{\sigma^2}\right)\right]$ is the capacity of the MIMO channel [1]. Since feedback does not increase the capacity of the memoryless channels, C_{mimo} is also the capacity of the MIMO channel with noiseless feedback, which serves as an upper bound on $R_{mimo}(n,\varepsilon)$.

[1] D. Tse, and P. Viswanath, Fundamentals of wireless communication, Cambridge university press, 2005.

Q2) Some errors, e.g., in Figure 1, the feedback signal Y_{i-1} should be Y^{i-1}, in line 133 of page 13, "channels" should be "channel", in Table I, the symbol $|\cdot|$ denotes modulus if applied to a complex number or cardinality if applied to a set, not only the cardinality of a set as the authors claim.

Answer: Thank you very much for the comment! We have tried our best to check through the manuscript and carefully corrected the issues mentioned above, and the revised Figure 1 is attached below.

Reply to Reviewer 3:

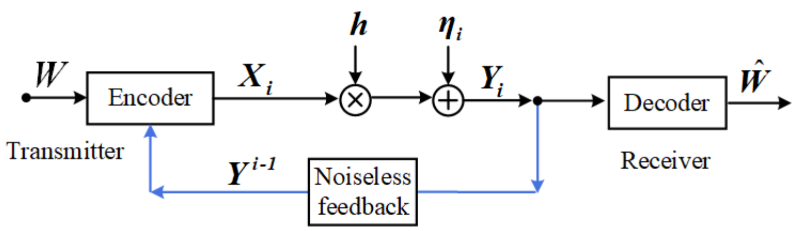

Fig. 1: The SISO/SIMO/MISO/MIMO channel with noiseless feedback link.

Q1) Authors have derived a converse bound for a special type of coding scheme in the finite blocklength regime. However, the results are claimed to show the exact channel capacity, which is not true. Therefore, I advise the authors to re-write their claims. On the other hand, several other converse bounds for finite blocklength regime coding with feedback have already been published in the literature. Thus, comparison with them is also important to understand if the proposed bound is loose or tight compared to the others.

Answer: Thank you very much for the comment! The main contribution of this paper is to provide the achievable bounds for SISO/SIMO/MISO/MIMO channel with noiseless feedback, which is based on a special class of linear feedback scheme known as the classical Schalkwijk-Kailath (SK) scheme, therefore, the derived achievable rate is expressed as the channel capacity minus the loss caused by the given codeword length and decoding error probability. To the best of the author's knowledge, the converse bound on the multi-antenna system with noiseless feedback remains open, and this is our future work.

Q2) Additionally, the inequality (a) in Eq. (20) is true for the asymptotic regime. Therefore, it is unclear if this inequality holds for finite blocklength. I advise the authors to revisit this issue and support their claims with additional references.

Answer: Thank you very much for the comment! The inequality (a) in Eq. (20) holds irrelevant to the finite blocklength, and the details are given below. The inequality (a) in Eq. (20) of the original manuscript is attached below (see Eq. (11)), which describes the decoding error probability analysis of the proposed SK-type coding scheme,

$$P_{e,R} \leq Pr\left\{|\varepsilon_{R,n}| \geq \frac{1}{2\cdot 2^{nR_R}}\right\} \overset{(a)}{\leq} 2Q\left(\frac{1}{2\cdot 2^{nR_R}\cdot\sqrt{\alpha_{R,n}}}\right), \tag{11}$$

where $Q(x)$ is the right-tailed function of the standard normal distribution and it's defined as $Q(x) = \frac{1}{\sqrt{2\pi}}\int_x^{\infty} e^{-\frac{t^2}{2}}dt$. Here inequality (a) is concretely expressed as

$$Pr\left\{|\varepsilon_{R,n}| \geq \frac{1}{2\cdot 2^{nR_R}}\right\} = Pr\left\{\{\varepsilon_{R,n} \geq \frac{1}{2\cdot 2^{nR_R}}\}\bigcup\{-\varepsilon_{R,n} \leq -\frac{1}{2\cdot 2^{nR_R}}\}\right\}$$

$$\leq 2Pr\left\{\varepsilon_{R,n} \geq \frac{1}{2\cdot 2^{nR_R}}\right\} \overset{(a1)}{=} 2Pr\left\{\varepsilon^{*}_{R,n} \geq \frac{1}{2\cdot 2^{nR_R}\cdot\sqrt{\alpha_{R,n}}}\right\}$$
$$\overset{(a)}{=} 2Q\left(\frac{1}{2\cdot 2^{nR_R}\cdot\sqrt{\alpha_{R,n}}}\right), \tag{12}$$

where (a1) follows from that $\varepsilon_{R,n} \sim \mathcal{N}(0, \alpha_{R,n})$ and $\varepsilon^{*}_{R,n} = \frac{\varepsilon_{R,n}-0}{\sqrt{\alpha_{R,n}}} \sim \mathcal{N}(0,1)$. Based on (12), we conclude that the inequality (a) holds irrelevant to the finite blocklength.

Q3) When perfect channel state is available at the transmitter and receiver, it is possible to match the rate with the instantaneous channel capacity. In this case, the maximum achievable capacity is the ergodic capacity, not log(1+SNR). However, Eq. (23) contradicts with this and claims that the achievable capacity is the instantaneous capacity. Are the Authors sure in their remark? If yes, please support your claims. Similar contradictions also appear in the other Remarks for SIMO, MISO, MIMO.

Answer: Thank you very much for the comment! We apologize for the unclear presentation of the assumptions in the original manuscript. In fact, the study of this paper is based on the assumptions that the channel state information (CSI) stays constant during the entire transmission and the CSI is perfectly known by the transceiver, therefore, the capacity given in Eq. (23) is the form of instantaneous capacity. The assumptions added in the revised manuscript is attached below.

Assumptions:

- We assume that all CSIs stay constant during the entire transmission.
- All CSIs are shared by the transceiver in the system.

Q4) I do not understand what is plotted in the Simulation Results. For instance, in Fig.2 there are two lines. One is the classical Shannon capacity log(1+SNR). What about the other? Is this the output of Eq. 6? If yes, it is also claimed that results are calculated based on an average of 1000 independent channel realizations. Therefore, it cannot be Eq. 6 right? If it is the average of achievable rates of the proposed codes, I believe it is more important to show the comparison between instantaneous channel vs the achieved rate with the proposed codes in time for several different blocklength?

Answer: Thank you very much for the comment! As the reviewer pointed out that there are two lines in Figure 2, one of which is the Shannon capacity and the other is the result given in Eq. (6) in original manuscript. Note that the channel coefficients stay constant during the entire transmission in the manuscript, and the statement “results are calculated based on an average of 1000 independent channel realizations” means that in MATLAB simulation, the channel coefficient is generated randomly, which causes the simulation curve is not smooth, in order to make the curve smoother, we generate 1000 channel coefficients and take the average value.

Q5) Wrong symbol in Eq. 3.

Answer: Thank you very much for the comment! We have tried our best to check through the manuscript and carefully corrected the issue mentioned above.

Finally, the authors would like to thank the editor and all the reviewers again for their valuable suggestions on improving this paper.
