# Supplementary material for: Finite blocklength feedback approach for multi-antenna wireless channels
Source: PLoS One. 2024 Feb 15;19(2):e0297810. doi: 10.1371/journal.pone.0297810 (PMC10868777; doi:10.1371/journal.pone.0297810)
Supplement: Attachment — Submitted filename: Response-to-Reviewer.pdf [file pone.0297810.s002.pdf]

Response to Referee Report on PONE-D-23-29974R1: *Finite Blocklength Feedback Approach for Multi-Antenna Wireless Channels*

Qiang Huang, and Tao Song

We would like to thank the editor and the reviewer again for the valuable comments and suggestions on improving this paper. We have addressed all the comments with utmost carefulness. The reply to editor and reviewer's comments is attached below.

Reply to Editor:

After careful consideration, we feel that it has merit but does not fully meet PLOS ONE's publication criteria as it currently stands. Therefore, we invite you to submit a revised version of the manuscript that addresses the points raised during the review process.

Answer: Thank you for giving us this chance to update our manuscript. Based on the reviewer's suggestions, the revision of this manuscript mainly includes:

- The significance and importance of Theorems 1-4 have been incorporated into the paper as a remark (see Remark 5) in the revised manuscript.
- As suggested by the reviewer, the non-math contents in math equations have been carefully corrected by using the *mathrm* command.
- In Introduction, the author name is mentioned at the reference number when mention a result. In addition, the additional space after the dash between two references has been deleted.

Reply to Reviewer:

Q1) The authors have addressed many of my comments. However, the reply to my comment 3 in the last round regarding the significance and importance of the results should be incorporated into the

paper as remarks. Furthermore, the subscript such as siso in h_{siso} should be written as h_{siso}. There are many such errors to be corrected throughout the paper. Finally, in the updated introduction, it would be good to mention author names instead of a reference number when mentioning a result and there is an additional space of citations of two papers of Hayashi.

Answer: Thank you very much for the comment!

- The significance and importance of the results have been incorporated into the paper as a remark (see Remark 5) in the revised manuscript, and for convenience, we attach it below.

  Remark 5. In the literature, the classical SK scheme has been shown to be a good finite blocklenth coding scheme for the AWGN channel with feedback since its decoding error probability doubly exponentially decays to zero as the coding blocklength tends to infinity. However, this well-performed scheme cannot be applied to practical wireless multi-antenna channels since it is only designed for the AWGN channel. In this paper, we extend the SK scheme to various wireless channels, and characterize the achievable transmission rates of these extended schemes (see Theorems 1-4), which provides a way for the application of the SK-type scheme to practical wireless communication systems. In addition, the established achievable rates in Theorems 1-4 can also be viewed as the fundamental limit of the achievable rates of the SK-type schemes for SISO/SIMO/MISO/MIMO channel with noisy feedback.
- The subscripts siso/simo/miso/mimo have been carefully corrected by using the *mathrm* command, and we have tried our best to check through the manuscript and carefully corrected such errors.
- As suggested by the reviewer, in Introduction, the author name is mentioned at the reference number when mention a result. In addition, the additional space after the dash between two references has been deleted.

Finally, the authors would like to thank the editor and all the reviewers again for their valuable suggestions on improving this paper.
