# Supplementary material for: Finite blocklength feedback approach for multi-antenna wireless channels
Source: PLoS One. 2024 Feb 15;19(2):e0297810. doi: 10.1371/journal.pone.0297810 (PMC10868777; doi:10.1371/journal.pone.0297810)
Supplement: Attachment — Submitted filename: Response-to-Reviewers.pdf [file pone.0297810.s003.pdf]

Response to Referee Report on PONE-D-23-29974R2: *Finite Blocklength Feedback Approach for Multi-Antenna Wireless Channels*

Qiang Huang, and Tao Song

We would like to express our sincere gratitude to the editor and all the reviewers again for the valuable comments and suggestions on improving this paper. The raised comments are very helpful for improving the quality of the manuscript. We have addressed all the comments with utmost carefulness. The reply to reviewers' comments is attached below.

Reply to Reviewer 1:

Q1) The non-math content in the figure legends should also be addressed. Pay attention to Fig. 2 to Fig. 5.

Answer: Thank you very much for the comment! The subscripts siso/simo/miso/mimo in Fig. 2 to Fig. 5 have been carefully corrected by using the *mathrm* command.

Q2) When citing papers, use et al. only if the number of authors is greater than three. Otherwise, list the family names of all authors.

Answer: Thank you very much for the comment! This kind of problems have been corrected.

Q3) The real and imaginary parts notations R and I are not math content as well. Thus, all subscripts involving these two should be addressed as well. The authors should check carefully for all such minor mistakes and correct them all.

Answer: Thank you very much for the comment! The subscripts R and I have been carefully corrected by using the *mathrm* command, and we have tried our best to check through the manuscript and carefully corrected such errors.

Reply to Reviewer 3:

Q1) I appreciate the revisions made to the manuscript. However, there still seems to be a discrepancy in the sections where the authors claim to be studying fading channels. The assumption that the channel remains constant throughout the entire communication and is known to both the transmitter and receiver, as indicated in the latest comments, seems to simplify the system model to a basic AWGN channel, which potentially limits the scope of the proposed method for fading channels. Therefore, I recommend that the authors consider a more cautious tone in presenting their contributions to fading channels. Hence, the authors should modify the abstract, contributions, and system model sections and avoid emphasizing that the study proposes a method for fading channels.

Answer: Thank you very much for the comment! Based on the reviewer's suggestion, in this revised manuscript, we modified the term "fading channel" to "quasi-static fading channel", which follows from the definitions mentioned in [1]-[2].

REFERENCES

[1] Karasik R, Simeone O, Renzo M D and Shamai Shitz S. Adaptive coding and channel shaping through reconfigurable intelligent surfaces: An information-theoretic analysis. IEEE Trans. Commun.. 2021 Nov; 69(11):7320-7334.

[2] Lin Y, Shen C, Hu Y, Ai B and Zhong Z. Joint design of channel training and data transmission for MISO-URLLC systems. IEEE Trans. Wireless Commun.. 2022 Oct; 21(10):8646-8659.

Finally, the authors would like to thank the editor and all the reviewers again for their valuable suggestions on improving this paper.
