# Supplementary material for: Finite blocklength feedback approach for multi-antenna wireless channels
Source: PLoS One. 2024 Feb 15;19(2):e0297810. doi: 10.1371/journal.pone.0297810 (PMC10868777; doi:10.1371/journal.pone.0297810)
Supplement: Attachment — Submitted filename: Response-to-Reviewer.pdf [file pone.0297810.s004.pdf]

Response to Referee Report on PONE-D-23-29974R3: *Finite Blocklength Feedback Approach for Multi-Antenna Wireless Channels*

Qiang Huang, and Tao Song

We would like to express our sincere gratitude to the editor and all the reviewers again for the valuable comments and suggestions on improving this paper. The raised comment is very helpful for improving the quality of the manuscript. We have addressed the comment with utmost carefulness. The reply to reviewer's comment is attached below.

Reply to Reviewer 3:

Q1) Thank you for addressing my comment and making revisions to the manuscript. However, I would like to point out that the term 'quasi-static fading channel' does not accurately represent the assumptions outlined in the manuscript. The assumption mentioned, where the channel remains constant throughout the entire communication and is known to both the transmitter and receiver, simplifies the system model to that of a basic Additive White Gaussian Noise (AWGN) channel. In a true quasi-static fading channel, while the channel conditions may change, they do so at a much slower rate compared to the transmission rate. Specifically, the channel coefficient is assumed to be an independent and identically distributed random variable according to some distribution but remains constant over the codeword transmission and it is unknown or partially known to the transmitter and receiver, which is also the case in the referenced papers. If my explanations remain unclear to the authors, I recommend reading Chapter 5.4 of David Tse's "Fundamentals of Wireless Communication" for further clarification. Hence, the mentioned assumption in the manuscript appears to lead to a scenario closer to a static channel rather than a quasi-static fading channel. Therefore, to maintain accuracy in the manuscript, it would be more appropriate to characterize the channel conditions as 'static channel' rather than using the term 'quasi-static fading channel'. I recommend revising the manuscript accordingly.

Answer: Thank you very much for the comment! We modified the term “quasi-static fading channel” to “static fading channel” in the revised manuscript.

Finally, the authors would like to thank the editor and all the reviewers again for their valuable suggestions on improving this paper.
