# Supplementary material for: Finite blocklength feedback approach for multi-antenna wireless channels
Source: PLoS One. 2024 Feb 15;19(2):e0297810. doi: 10.1371/journal.pone.0297810 (PMC10868777; doi:10.1371/journal.pone.0297810)
Supplement: Attachment — Submitted filename: Response-to-Reviewer.pdf [file pone.0297810.s005.pdf]

Response to Referee Report on PONE-D-23-29974R4: *Finite Blocklength Feedback Approach for Multi-Antenna Wireless Channels*

Qiang Huang, and Tao Song

We would like to express our sincere gratitude to the editor and all the reviewers again for the valuable comments and suggestions on improving this paper. The raised comment is very helpful for improving the quality of the manuscript. We have addressed the comment with utmost carefulness. The reply to reviewer's comment is attached below.

Reply to Reviewer 3:

Q1) Once again, I would like to highlight that the term 'static fading channel' is still misleading. In wireless communication, a 'fading channel' typically refers to a scenario where the channel conditions vary over time, and using the term 'static' creates confusion. In fact, there is no such thing as 'static fading channel' in wireless communication. On the other hand, the term 'static fading channel' is still but very rarely used in the literature, however, when used, it consistently refers to quasi-static channels. Therefore, if the intention is to convey that the channel conditions remain constant during the entire communication, which is the case in the current manuscript, the term 'static' alone without 'fading' is accurate.

Answer: Thank you very much for the comment! We modified the term "static fading channel" to "static channel" in the revised manuscript.

Finally, the authors would like to thank the editor and all the reviewers again for their valuable suggestions on improving this paper.
